# Ototoxicity from Combined Cisplatin and Radiation Treatment: An In Vitro Study

**DOI:** 10.1155/2010/523976

**Published:** 2010-12-01

**Authors:** Wong-Kein Low, Sylvia W. W. Kong, Michelle G. K. Tan

**Affiliations:** ^1^Department of Otolaryngology, Singapore General Hospital, Singapore 169608; ^2^Department of Clinical Research, Singapore General Hospital, Singapore 169608

## Abstract

*Objective*. Combined cisplatin (CDDP) and radiotherapy is increasingly being used to treat advanced head and neck cancers. As both CDDP and radiation can cause hearing loss, it is important to have a better understanding of the cellular and molecular ototoxic mechanisms involved in combined therapy. *Procedure*. The effects of CDDP, radiation, and combined CDDP-radiation on the OC-k3 cochlear cell line were studied using MTS assay, flow cytometry, Western blotting, and microarray analysis. *Results*. Compared to using CDDP or radiation alone, its combined use resulted in enhanced apoptotic cell death and apoptotic-related gene expression, including that of FAS. Phosphorylation of p53 at Ser15 (a marker for p53 pathway activation in response to DNA damage) was observed after treatment with either CDDP or radiation. However, posttreatment activation of p53 occurred earlier in radiation than in CDDP which corresponded to the timings of MDM2 and TP53INP1 expression. *Conclusion*. Enhanced apoptotic-related gene expressions leading to increased apoptotic cell deaths could explain the synergistic ototoxicity seen clinically in combined CDDP-radiation therapy. CDDP and radiation led to differential temporal activation of p53 which suggests that their activation is the result of different upstream processes. These have implications in future antiapoptotic treatments for ototoxicity.

## 1. Introduction

Combined chemoradiotherapy is increasingly being used to treat advanced head and neck cancers. During radiotherapy, the ear structures are often included in the radiation fields and it is generally accepted that radiation-induced sensorineural hearing loss can result. Cisplatin (CDDP), widely used as an effective antineoplastic drug for these cancers, is also known to cause ototoxicity. In a randomized blinded study, it was demonstrated that patients who had received radiotherapy and concurrent/adjuvant chemotherapy using CDDP experienced greater sensorineural hearing loss compared with patients treated with radiotherapy alone [1]. This was especially so in the high-frequency sounds of the speech range, resulting in significant hearing disability. 

In recent years, immortalized cell lines derived from the mouse organ of Corti had been developed and characterized [2]. For example, the OC-k3 cell line was derived from the organ of Corti of the transgenic mouse. It encoded the large T antigen of the SV40 (simian virus 40), a thermolabile viral protein which drove the cells to proliferate indefinitely at 33°C and in the presence of gamma interferon [3]. This cell line expressed the neuro-epithelial precursor cell marker nestin and the inner ear cell marker OCP2, but did not exhibit markers for glial or neuronal cells. In addition, OC-k3 cells expressed specific auditory sensory cell markers (myosin VIIa and the acetylcholine receptor alpha-9) and the supporting cell marker connexin 26. This and other similar cell lines had been regarded as good models to study the mechanisms of cell fate in the organ of Corti of the cochlea [4]. 

P53 had been found to play an important role in apoptotic cell death associated with ototoxicity. In a CDDP-induced apoptosis experiment using cochlear organotypic cultures prepared from rats at postnatal days 3-4, significant upregulation of phospho-p53 serine 15 expression was found and apoptosis was suppressed by pifithrin-*α*, a p53 inhibitor [5]. Other studies have shown that the deletion of the p53 gene protects sensory hair cells from CDDP-induced cell death, caspase-2 activation, and cytochrome c translocation [6]. In radiation-induced ototoxicity, it was found that p53 together with reactive oxidative species (ROS) played an important role in cochlear cell apoptosis [7]. 

In the combined use of CDDP and radiation, the cellular and molecular mechanisms leading to ototoxicity had not been studied. It is important to have a better understanding of these mechanisms as effective preventive strategies directed at the relevant pathways can potentially be developed. The present study found that although p53 played a role in both CDDP and radiation-induced cochlear cell apoptosis, p53 was activated at different time points after each treatment which corresponded to the time MDM2 and TP53INP1 were expressed. Additional apoptotic-related genes that were not expressed when CDDP or radiation was used alone were expressed when used in combination. This included FAS, an important element involved in the extrinsic apoptotic pathway.

## 2. Materials and Methods

### 2.1. Cell Culture

The immortalized OC-k3 cell line derived from the organ of Corti of the transgenic mice (Immortamouse H-2Kb-tsA58, Charles Rivers Laboratories, Wilmington, MA) was used. The cell line was cultured in high-glucose Dulbecco's Eagle's medium (DMEM, Gibco, Grand Island, NY) supplemented with 10% fetal bovine serum (FBS, Gibco, Grand Island, NY), 1% penicillin-streptomycin (P/S, Gibco, Grand Island, NY), and 50 U/ml gamma-interferon (mouse recombinant, Sigma-Aldrich, St. Louis, MO) and maintained at 33°C with 10% CO_2_. To study the impact of chemoradiation treatment, OC-k3 cells were exposed to 5 Gy of gamma irradiation alone, 0.5 *μ*g/ml of cisplatin alone, or 5 Gy of gamma irradiation in the presence of 0.5 *μ*g/ml cisplatin (Pfizer, Bentley, WA).

### 2.2. Cell Viability Assay

The OC-k3 cells were seeded in 96-well plates at densities of 5 × 10^3^ cells/well in 200 *μ*l complete medium after being exposed to chemo-irradiation treatment. Cell viability was determined using CellTiter 96 Aqueous One Solution Cell Proliferation Assay (Promega Corp., Madison, WI) containing tetrazolium compound 3-[4,5-dimethylthiazol-2-yl]-5-(3-carboxymethoxyphenyl)-2-(4-sulfophenyl)-2H-tetrazolium (MTS) at 3 h, 24 h, 48 h, and 72 h after chemo-irradiation. This test was based on the bioreduction of MTS compound into a soluble and colored formazan product by NADPH or NADH, which is produced by dehydrogenase enzymes in metabolically active cells. Twenty microliters of MTS were added to each well, incubated at 33°C for 3 h, and then the absorbance was recorded at 490 nm with a microplate spectrophotometer (Benchmark Plus, Bio-Rad Laboratories, Hercules, CA).

### 2.3. Cell Death Analysis

The cells were collected at each time point post CDDP-radiation treatment, fixed in 75% ethanol and stored at 4°C. Upon analysis, the cells were washed with PBS and incubated with 100 *μ*g/ml propidium iodide (PI) containing 0.1% Triton X-100 and 500 *μ*g/ml RNase A in 50 *μ*l PBS for 30 mins in darkness at 4°C. The DNA contents of cells were analyzed using the flow cytometer CyAnTM ADP Analyser (Beckman Coulter, Fullerton, CA). The magnitudes of the sub-G1 fractions were determined using the Summit 4.3 software (Beckman Coulter, Fullerton, CA). DNA fragmentation resulting from apoptotic cell death would manifest in the sub-G1 fraction.

### 2.4. Western Blot Analysis

Protein extraction was done by incubating the cells at 4°C for 30 minutes in lysis buffer containing 150 mM NaCl, 10 mM Tris-HCl pH 7.4, 2 mM EDTA, 0.5 mM EGTA, 1 mM sodium orthovanadate, 0.1% sodium deoxycholate, 0.5% NP-40, and 1% Triton X-100 supplemented with 1x complete protease inhibitor mixture (Roche, Basel, Switzerland). Equal amounts of protein samples were denatured separated by 10% SDS-PAGE and transferred onto nitrocellulose membrane by iBlot dry blotting system (Invitrogen, Carlsbad, CA). The membrane was blocked with 5% nonfat milk in PBS with 0.1% Tween-20 (PBST) for 1 h, followed by an overnight incubation of primary antibodies in 5% BSA/PBST at 4°C. Primary antibodies included anti-p53 pAb (NCL-p53-CM5p, Novocastra), anti-phospho-p53 (ser-15) pAb, anti-phospho-c-jun (ser-73) pAb, anti-c-jun (60A8) mAb (Cell Signaling Technology, Inc.), and anti-beta-actin mAb (Sigma-Aldrich, St. Louise, MO). After washing the membrane extensively, incubation with horseradish peroxidase-conjugated antirabbit or antimouse secondary antibody (Cell Signaling Technology, Inc.) was done for 1 h at room temperature. After washing, the membrane was incubated in Immobilon Western chemiluminescent HRP substrate (Millipore, Billerica, MA), and the chemiluminescence signals were detected using UVIchemi (UVItec, Cambridge, UK), a dedicated chemiluminescence documentation system. For reprobing with a new primary antibody, the membrane was stripped in Re-Blot plus strong solution Western blot stripping buffer (Chemicon, Temecula, CA) at room temperature for 30 minutes and rinsed 3 times with PBST for 10 minutes each time.

### 2.5. Microarray Analysis

The global changes of gene expression were analyzed at 3 h, 24 h, and 72 h after chemoirradiation, on the GeneChip Mouse Genome 430A 2.0 Array (Affymetrix, Santa Clara, CA). Biological duplicates of experiments were performed. Briefly, RNA was extracted from cells using TRIzol reagent (Invitrogen, Carlsbad, CA) followed by generation of double-stranded cDNA. These were used as templates for synthesis of biotin-labeled cRNA, using the GeneChip IVT labeling kit in accordance with the manufacturer's instructions. The biotinylated cRNA was purified using RNeasy Mini kit (Qiagen, Hilden, Germany) and fragmented before reconstitution in a hybridization cocktail mixture containing eukaryotic hybridization control. The hybridization was performed at 45°C for 16 h in a rotisserie oven set at 60 rpm. Upon completion, the arrays were then loaded onto an Affymetrix Fluidic station, washed according to the standard Affymetrix EukGE-WS2v5 protocol and stained with streptavidin-phycoerythrin (SAPE) solution. After washing and staining, the arrays were scanned with the Gene Array scanner (Affymetrix, Santa Clara, CA). Hybridization intensity data detected by the scanner were automatically acquired and processed by the Affymetrix GeneChip Operating Software (GCOS, Affymetrix, Santa Clara, CA). The average intensity for all the genes was normalized to 100. The statistical algorithms implemented in GCOS software were used for analysis. In a comparison expression analysis, each probe pair on the experimental array was compared to the corresponding probe pair on the baseline array (control). This generated an associated “change” (increased, no change, or decreased) to determine the relative expression of transcripts. To have an overview of gene expression profiles, probe sets showing chemoradiation-induced increased or decreased expressions in both duplicated experiments were retrieved. The differentially expressed genes of chemoradiation treatment were submitted for biological functional analysis using Ingenuity Pathway Analysis (IPA) tools (Ingenuity Systems, http://www.ingenuity.com).

## 3. Results

### 3.1. Combined CDDP-Radiation Treatment Reduced Cell Viability More than CDDP or Radiation Treatment Alone

**Figure 1:**
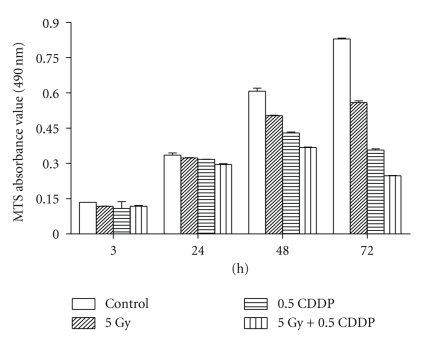
Cell viability analysis by MTS assay at different time points (3 h, 24 h, 48 h, and 72 h) after treatment with 5 Gy gamma radiation and 0.5 *μ*g/ml cisplatin (CDDP). After co-treatment with radiation and CDDP, cell viability was significantly reduced at 72 h. The data shown are the most representative of 3 separate experiments.

Cell viability analysis by MTS assay at different time points revealed that although CPPD and radiation each exerted a negative effect on cell viability, treatment when combined appeared to have a greater effect. These effects were observed at 48 hrs after treatment and became even more marked at 72 hrs after treatment (Figure 1).

### 3.2. Apoptosis Occurred Predominantly at 72 h after Combined CDDP-Radiation Treatment

**Figure 2:**
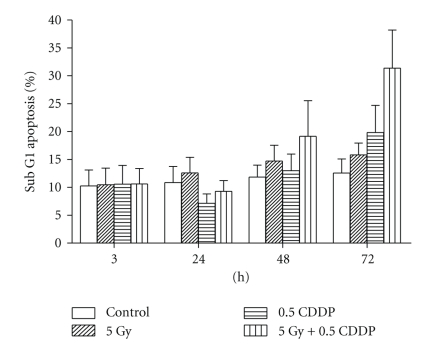
Flow cytometric subG1 phase as determined by PI staining at different time points (3 h, 24 h, 48 h and 72 h) after exposure to 5 Gy of gamma radiation and 0.5 *μ*g/ml of cisplatin (CDDP). Co-treatment with radiation and CDDP resulted in a significant increase in subG1 phase at 72 h. The data shown are the mean + SD of 4 independent experiments.

At 72 hrs after treatment, combined CDDP-radiation led to a greater increase in the sub-G1 phase as compared to using CDDP and radiation alone (Figure 2). As pointed out previously, DNA fragmentation resulting from apoptotic cell death manifests in the sub-G1 fraction.

### 3.3. Apoptosis-Related Gene Expressions were Enhanced by Combined CDDP-Radiation Treatment

**Figure 3:**
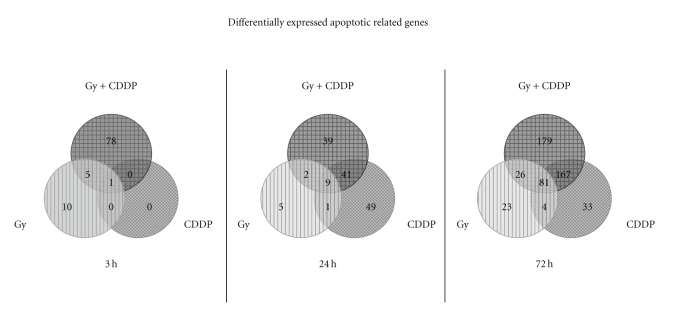
Microarray findings are summarized by Venn diagrams which show the distribution of differentially expressed probeset IDs in each treatment group [irradiation (Gy), cisplatin (CDDP) or combination of both (Gy and CDDP)] when compared to nontreated control cells at 3 h, 24 h, and 72 h after treatment.

On analyzing the results of molecular and cellular functions under the biological functions of IPA, it was found that among the 3925 probe set IDs which were differentially expressed in at least one treatment, 942 represented 623 unique genes associated with apoptosis (see Table 1). Their distribution at each time point for the different treatment regimes is summarized in Venn diagrams (Figure 3). A subset focusing on the genes, which had a direct upstream or downstream relationship with p53, is shown in Table 2. Combined CDDP-radiation treatment resulted in an increase in the number of gene expressions which was more than merely a summation of the number of expressions resulting from individual treatments (Figure 3, Table 2). At 72 hrs after treatment, 40 out of the 163 genes listed (24.5%) were expressed in combined CDDP-radiation treatment, but not when CDDP or radiation was used alone (Table 2). Among these 40 genes was FAS, an important element of the extrinsic apoptotic pathway.

### 3.4. Differential Temporal Activation of p53 Occurred with CDDP and Radiation Treatment

**Figure 4:**
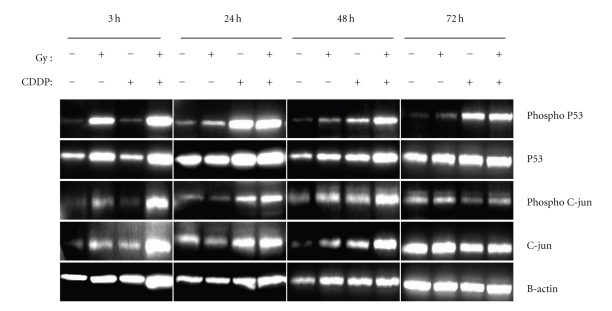
Western blot analysis showing p53 and c-jun protein expression and phosphorylation at various time points (3 h, 24 h, 48 h, and 72 h) after 5 Gy of gamma radiation and 0.5 *μ*g/ml pf cisplatin (CDDP). The data are representative of 3 separate experiments.

It was observed that Posttreatment activation of p53 occurred earlier in radiation than in CDDP (Figure 4). In response to DNA damage, activation of the p53 pathway normally occurs with the phosphorylation of ser-15 in p53. The present study showed radiation-induced phosphorylation of p53 occurred at 3 hrs after treatment, compared to CDDP-induced activation which was observed only at 24 hrs or later (Figure 4). These timings corresponded with those observed for the expression of apoptotic-related genes after radiation and CDDP treatment (Figure 3). For example, MDM2 and TP53INP1 were expressed at 3 hrs after radiation. They were however, expressed only at 24 hrs after CDDP (Table 2).

## 4. Discussion

Combined chemoradiation is increasingly being used to treat advanced head and neck caners. As radiation and CDDP are both ototoxic, it is of concern that significant sensorineural hearing loss will result. Indeed, patients with nasopharyngeal carcinoma who had received radiotherapy and concurrent/adjuvant chemotherapy using CDDP were found to experience greater sensorineural hearing loss compared with patients treated with radiotherapy alone, especially to high-frequency sounds in the speech range [1]. It is of interest to note that different etiologies of sensorineural hearing loss, such as noise, ototoxic drugs, and aging, result in similar patterns of audiometric changes and cochlear cellular degeneration [8]. The cellular and molecular mechanisms involved in sensorineural hearing loss from diverse causes appear to lead to a final common pathway which results in apoptosis of cochlear hair cells [6, 9].

In radiation-induced ototoxicity, cochlear cell apoptosis and ROS generation were observed after irradiation, and p53 was thought to play a key role [7]. This phenomenon was dose dependant and occurred predominantly at 72 h after irradiation. Microarray analysis supported these findings, as associated dose-dependant apoptotic gene regulation changes were observed. 

The ototoxic manifestations of CDDP are primarily due to its effects on the cochlear hair cells although the spiral ganglion cells and the stria vascularis are also affected to some extent. According to Rybak et al. [10], CDDP ototoxicity appears to be triggered by ROSs that initiate a cascade of molecular events that lead to apoptosis of outer hair cells, resulting in hearing loss. Ototoxic effects on the stria vascularis are transient, resulting in temporary reduction of endocochlear potential associated with stria edema. The endocochlear potential recovers but residual shrinkage of the strial persists. The spiral ganglia are thought to be least affected. 

Although the cellular and molecular processes of ototoxicity have been described for radiation and CDDP when used alone, those involved in combined therapy have not been studied previously. The present study demonstrated that combined therapy led to decreased viability of cochlear cells, with an increase in the subG1 population. These findings support the belief that as in other etiologies of sensorineural loss, apoptosis of cochlear hair cells is important in CDDP-radiation.

It is well established that p53 plays a key role in the cellular response to nuclear DNA damage [11]. It regulates cell cycle arrest and dictates cell fate like senescence, apoptosis, and DNA repair. It is believed that the nature of DNA damage enables p53 to selectively discriminate between promotors in the induction of target genes, thereby regulating their expression and subsequent cellular outcome [12]. 

In a study on HEI-OC1 cells derived from the cochlea, CDDP caused an increase in p53 at 3 hrs prior to the activation of Bax, cytochrome-c, and caspase 8 and 9 [13]. In the case of radiation-induced ototoxicity, the role of p53 in triggering apoptotic cell death in cochlear hair cells has also been studied [7]. Based on microarray analysis, the p53 gene was found to be up-regulated after irradiation and p53 expression was confirmed by Western blotting. Although p53 plays a role in both CDDP and radiation-induced ototoxicity, the present study showed that p53 was activated at different time points after treatment. Posttreatment phosphorylation of p53 occurred after 24 hrs for CDDP, whereas it occurred as early as 3 hrs for radiation. These timings corresponded to the times MDM2 and TP53INP1 were expressed after treatment with CDDP and radiation respectively. Therefore, although both CDDP and radiation-induced cochlear cell apoptosis appear to involve activation of p53, the upstream processes involved may well be different.

In the present study, combined CDDP-radiation treatment triggered more apoptotic-related gene expressions than those that could be accounted for by a summation of gene expressions resulting from individual treatments. This could explain the synergistic ototoxic effects of combined CDDP-radiation treatment, an observation seen clinically [1]. Interestingly, among the genes which were expressed in combined treatment but not when these entities were used alone was FAS, a key element involved in the extrinsic apoptotic pathway. Although the extrinsic apoptotic pathway has generally been regarded to play only minor role in ototoxicity resulting from the use of CDDP or radiation alone, it may well be important in situations when they are used in combination [14, 15]. 

The OC-k3 cell line expressed the neuroepithelial precursor cell marker nestin and the inner ear cell marker OCP2, specific auditory sensory cell markers myosin VIIa and the acetylcholine receptor alpha-9 and the supporting cell marker connexin 26. It had been regarded as a good model to study the mechanisms of cell fate in the Organ of Corti of the cochlea [4]. Therefore, the finding that combined treatment actually led to enhanced apoptotic gene expressions including FAS should be further investigated in *in vivo* animal studies which may have implications in future antiapoptotic treatments against ototoxicity.

## 5. Conclusion

Like in other etiologies of sensorineural loss, apoptosis of cochlear hair cells appears to play a role in ototoxicity resulting from combined CDDP-radiation therapy. Differential temporal activation of p53 suggests the possibility of different upstream processes leading to its activation after CDDP and radiation treatment. Enhanced apoptotic gene expressions including that of FAS were observed in combined treatment which could possibly explain the synergistic ototoxic effects seen clinically.

## Figures and Tables

**Table 1:** Differentially expressed apoptosis-related genes in each treatment group [irradiation (Gy), cisplatin (CDDP), or combination of both (Gy + CDDP)] when compared to nontreated control cells at 3 h, 24 h, and 72 h after treatment

		3 h			24 h			72 h		

Symbol	Probe set ID	Gy	CDDP	Gy + CDDP	Gy	CDDP	Gy + CDDP	Gy	CDDP	Gy + CDDP
AAK1	1420025_s_at, 1434935_at								I	I
AARS	1423685_at									D
ABCB1B	1418872_at								I	I
ABCC1	1452233_at									I
ABCG2	1422906_at									I
ABL2	1455495_at									I
ACSL4	1451828_a_at									I
ACTN4	1423449_a_at									I
ACVR1	1448460_at					D				
ADAMTSL4	1451932_a_at									I
ADM	1416077_at, 1447839_x_at					D	D	I		I
AES	1420619_a_at						D			
AFP	1416645_a_at									I
AHR	1422631_at									I
AIMP1	1416486_at									D
AKAP12	1419706_a_at								I	I
AKT1S1	1428158_at, 1452684_at									D
ALDH1A1	1416468_at								I	I
ALDH1A2	1422789_at									D
ALDOA	1416921_x_at, 1433604_x_at, 1434799_x_at, 1439375_x_at					D	D	I	I	I
ANKRD1	1420991_at, 1420992_at							I	I	I
ANLN	1433543_at, 1439648_at								I	I
ANP32A	1421918_at					D				
ANXA1	1448213_at							I	I	I
ANXA7	1416138_at								I	I
AP2A2	1452490_a_at									I
APBB2	1452342_at									I
APEX1	1416135_at, 1437715_x_at, 1456079_x_at					D	D			
APOE	1432466_a_at							I	I	I
APPL1	1455159_at					I				
ARNT2	1434028_at									I
ASAH2	1450726_at								I	I
ATF3	1449363_at			D				D		D
ATF5	1425927_a_at							D		D
ATG12	1451746_a_at								D	D
ATG5	1418235_at			I						
ATM	1421205_at, 1428830_at								I	I
ATN1	1421149_a_at								I	
ATP1A1	1451071_a_at						D			
ATP7A	1436921_at					I				
ATXN2	1460653_at			D						
AURKA	1424511_at			D				I	I	I
AXL	1423586_at			D					I	I
BAG3	1422452_at			D						
BANF1	1421081_a_at, 1421082_s_at, 1421083_x_at								D	D
BCAR1	1439388_s_at, 1450622_at			D					I	I
BCL3	1418133_at							I		I
BCLAF1	1428844_a_at, 1428845_at, 1436023_at, 1438089_a_at				I	I				
BDNF	1422168_a_at									I
BECN1	1455880_s_at, 1460320_at								I	I
BGN	1437889_x_at, 1448323_a_at					D	D			I
BHLHE40	1418025_at					D	D			I
BID	1417045_at, 1448560_at			D					I	I
BIRC3	1421392_a_at									I
BIRC5	1424278_a_at								I	I
BLM	1448953_at					I				
BNIP2	1453993_a_at									D
BNIP3	1422470_at							D		
BPTF	1427310_at, 1456615_a_at					I				
BRAF	1435434_at					I				
BRCA1	1424629_at, 1424630_a_at, 1451417_at					I				
BRD2	1423502_at, 1437210_a_at					D	D			D
BRE	1426312_at, 1426313_at					D	D			D
BTG1	1426083_a_at					D				
BTG2	1416250_at, 1448272_at	I					I			
BUB1	1424046_at					I	I		I	I
BUB1B	1416961_at, 1447363_s_at							I	I	I
C11ORF82	1429734_at			I			I			I
C1QBP	1455821_x_at								D	
C3	1423954_at							I		I
CACNA1A	1450510_a_at								I	
CACNA1C	1421297_a_at								D	D
CASP12	1449297_at							D		
CASP2	1448165_at							D		
CASP3	1426165_a_at, 1449839_at			I						
CASP6	1415995_at			I						I
CASP7	1426062_a_at, 1448659_at							D		D
CASP9	1426125_a_at	D								
CAST	1426098_a_at, 1435972_at, 1451413_at					I	I	I	I	I
CAT	1416429_a_at									I
CAV1	1449145_a_at					D		I	I	I
CBX5	1421933_at, 1450416_at							D		D
CCAR1	1436156_at, 1436157_at					I	I			
CCL13	1420380_at						I	I	I	I
CCL5	1418126_at				I		I		I	I
CCL9	1417936_at, 1448898_at								I	I
CCNA2	1417910_at, 1417911_at								I	I
CCNB1	1416076_at, 1419943_s_at, 1448205_at, 1449675_at			D				I	I	I
CCND1	1417419_at, 1417420_at, 1448698_at	D						I	I	I
CCND3	1415907_at								I	I
CCNG1	1420827_a_at, 1450016_at, 1450017_at	I		I	I	I	I	I	I	I
CD14	1417268_at									I
CD24	1416034_at, 1437502_x_at, 1448182_a_at			D						I
CD274	1419714_at						I	D		
CD2AP	1420907_at					I				
CD44	1423760_at, 1434376_at, 1452483_a_at								I	I
CD47	1419554_at, 1428187_at, 1449507_a_at							D		D
CD80	1432826_a_at									I
CD9	1416066_at			D				I	I	I
CDC20	1416664_at, 1439377_x_at							I	I	I
CDC25B	1421963_a_at								I	I
CDC25C	1422252_a_at, 1456077_x_at								I	I
CDC2L2	1418841_s_at									D
CDC37	1416819_at								I	I
CDC42EP3	1422642_at, 1450700_at							I	I	I
CDC45L	1416575_at							D		D
CDC6	1417019_a_at									D
CDCA2	1437251_at, 1455983_at								I	I
CDH2	1418815_at									I
CDK4	1422439_a_at, 1422440_at, 1422441_x_at						D		D	D
CDK8	1460389_at									I
CDKN1A	1421679_a_at, 1424638_at	I		I	I	I	I	I	I	I
CDKN1B	1434045_at								D	D
CDKN2A	1450140_a_at						D			
CDKN2C	1416868_at						D			
CEBPB	1427844_a_at			D		D				
CEBPD	1423233_at					D		I	I	I
CENPF	1427161_at									I
CFLAR	1424996_at									I
CHEK1	1439208_at									D
CKAP2	1434748_at					I	I		I	I
CLCF1	1437270_a_at, 1450262_at			D						I
CLU	1418626_a_at, 1437458_x_at, 1437689_x_at, 1454849_x_at							I	I	I
CNN2	1450981_at								I	I
CNP	1418980_a_at, 1437341_x_at								I	I
CNTF	1426327_s_at									D
COPS5	1460171_at									D
CR1	1422563_at									D
CREB3L1	1419295_at								I	I
CRK	1416201_at, 1448248_at			D			I		I	I
CROP	1424802_a_at, 1451485_at			I		I				D
CRYAB	1416455_a_at, 1434369_a_at			I					I	I
CSF1	1425154_a_at, 1425155_x_at, 1448914_a_at, 1460220_a_at			D					I	I
CSF2	1427429_at									I
CSNK2A1	1419034_at, 1419035_s_at, 1419036_at, 1419038_a_at								D	D
CST3	1426195_a_at							I		
CTCF	1418330_at, 1449042_at	D								D
CTGF	1416953_at							I	I	I
CTNNA1	1437807_x_at, 1448149_at									I
CTSB	1417490_at, 1417491_at, 1417492_at								I	I
CTSD	1448118_a_at								I	I
CTTN	1421313_s_at, 1421315_s_at, 1423917_a_at, 1433908_a_at								I	I
CUL3	1434717_at							D		
CUL5	1428287_at					I				
CX3CL1	1415803_at									I
CXCL12	1417574_at, 1448823_at					D	D			
CXCL2	1419209_at, 1441855_x_at, 1457644_s_at					D		I	I	I
CXCR7	1417625_s_at					D	D		I	I
CYB5A	1416727_a_at									I
CYB5R3	1422185_a_at, 1422186_s_at, 1425329_a_at								I	I
CYBA	1454268_a_at								I	
CYLD	1429617_at									I
CYR61	1416039_x_at, 1438133_a_at, 1442340_x_at, 1457823_at							I	I	I
DAB2	1420498_a_at, 1423805_at, 1429693_at								I	I
DAP	1423790_at, 1451112_s_at					D	D	I	I	I
DAXX	1419026_at							D	I	
DCN	1449368_at									I
DDIT3	1417516_at									D
DDIT4	1428306_at							I	I	I
DDR1	1415797_at, 1415798_at, 1456226_x_at			D					I	I
DDX5	1419653_a_at	I								
DDX58	1436562_at, 1456890_at							D	I	D
DHCR24	1451895_a_at								I	I
DKK3	1417312_at, 1448669_at					D	D	I	I	I
DLC1	1436173_at, 1460602_at									I
DLX2	1448877_at					D				
DNAJC15	1416910_at									D
DNM1L	1428086_at, 1452638_s_at					I				
DTYMK	1438096_a_at			I						
DUSP14	1431422_a_at								I	I
DUSP22	1448985_at								I	
DUSP4	1428834_at			D						
DUSP6	1415834_at									I
DUT	1419270_a_at									D
E2F1	1417878_at							D		
ECOP	1451127_at							D		
EDA2R	1440085_at	I		I				I	I	I
EEF1D	1439439_x_at, 1449506_a_at								D	D
EGR1	1417065_at							I	I	I
EHD4	1449852_a_at								I	
EIF2AK2	1422006_at, 1440866_at				I	I	I			
EIF4E	1450908_at									D
EIF5A	1437859_x_at									D
ELAVL1	1452858_at									D
EMILIN2	1435264_at								I	I
EMP1	1416529_at							I	I	I
EMP3	1417104_at							I	I	I
ENO1	1419022_a_at, 1419023_x_at							I		
EPHA2	1421151_a_at									I
EPHX1	1422438_at							I	I	I
ERCC3	1448497_at									I
ERCC5	1450935_at									I
ESPL1	1433862_at									I
ETS1	1422027_a_at, 1426725_s_at, 1452163_at			D						I
ETS2	1416268_at							D		
EVI1	1438325_at					D	D			
EWSR1	1417238_at								I	I
EXOC2	1428470_at									I
EZR	1450850_at								I	I
F2R	1437308_s_at, 1450852_s_at									I
F3	1417408_at						D			
FAS	1460251_at									I
FASN	1423828_at									I
FBL	1416684_at, 1416685_s_at								D	D
FBN1	1425896_a_at, 1438870_at, 1460208_at								I	I
FDFT1	1438322_x_at, 1448130_at						D		I	I
FEN1	1421731_a_at, 1436454_x_at							D	D	D
FGF7	1422243_at, 1438405_at							I	I	I
FGFR1	1424050_s_at			D					I	I
FHL2	1419184_a_at			D					I	I
FKBP1B	1449429_at								I	I
FLT3LG	1422115_a_at								I	
FN1	1437218_at									I
FOS	1423100_at								I	I
FOSL1	1417487_at, 1417488_at							I	I	I
FOXM1	1417748_x_at, 1448833_at, 1448834_at, 1453107_s_at								I	I
FOXO1	1416982_at						D			
FOXP1	1421141_a_at, 1421142_s_at, 1435222_at						D			
FST	1421365_at, 1434458_at				I		I	I		I
FSTL1	1416221_at, 1448259_at							I	I	I
FTH1	1427021_s_at							I		
FUBP1	1433482_a_at, 1433640_at						I		D	D
FUS	1451285_at, 1455831_at			I				D		
FXN	1427282_a_at									D
FXR1	1417598_a_at, 1442059_at, 1452247_at					I				
FYN	1448765_at									I
G2E3	1434699_at, 1455355_at								I	I
G6PD	1448354_at								I	I
GABPA	1450665_at									D
GADD45A	1449519_at							D	D	D
GAS1	1416855_at, 1448494_at	D			D	D	D		D	D
GATAD2A	1423992_at, 1451197_s_at, 1451198_at, 1455505_at			D						I
GDF15	1418949_at	I							I	
GDNF	1419080_at									I
GFRA1	1450440_at								I	I
GHR	1417962_s_at, 1451501_a_at								I	I
GJA1	1415800_at, 1415801_at, 1437992_x_at, 1437992_x_at, 1438650_x_at, 1438945_x_at, 1438973_x_at					D	D	I		I
GLIPR1	1424927_at							I	I	I
GLRX	1416592_at, 1416593_at								I	I
GNA12	1421026_at, 1450097_s_at									D
GNA13	1422556_at, 1433749_at, 1450656_at, 1453470_a_at, 1460317_s_at			D				D		
GNPNAT1	1423158_at									D
GPI	1420997_a_at, 1434814_x_at, 1450081_x_at									I
GPX1	1460671_at									I
GRN	1448148_at								I	
GSK3B	1437001_at, 1451020_at, 1454958_at								D	D
GSN	1415812_at, 1436991_x_at, 1437171_x_at, 1456312_x_at								I	I
GSPT1	1426736_at, 1452168_x_at									D
GSTM1	1416411_at								I	
GSTM5	1448330_at						D			
HBEGF	1418349_at			D						
HELLS	1417541_at					I				
HIP1	1434557_at			D						
HIPK1	1424540_at			D						
HIST1H1C	1416101_a_at, 1436994_a_at					D	D	I	I	
HK1	1420901_a_at								I	I
HK2	1422612_at									I
HMGA1	1416184_s_at							I	I	I
HMGA2	1422851_at, 1450780_s_at, 1450781_at							I	I	I
HMGB1L1	1425048_a_at, 1435324_x_at, 1439463_x_at, 1448235_s_at								D	D
HMGN1	1455897_x_at								D	
HMMR	1425815_a_at, 1427541_x_at, 1450156_a_at, 1450157_a_at					I			I	I
HMOX1	1448239_at									D
HNRNPA1	1423531_a_at, 1430019_a_at, 1430020_x_at							D	D	D
HOXA7	1449499_at									D
HSH2D	1442130_at								I	I
HSP90AA1	1426645_at, 1437497_a_at, 1438902_a_at			I		I	I			
HSP90AB1	1416364_at, 1416365_at									I
HSPA1B	1427127_x_at						D			
HSPA5	1416064_a_at, 1427464_s_at, 1447824_x_at								D	D
HSPB1	1422943_a_at, 1425964_x_at			D				I	I	I
HSPB8	1417014_at									D
HTATIP2	1451814_a_at									I
HUWE1	1415703_at									D
ID1	1425895_a_at									I
ID2	1422537_a_at						D			
IER3	1419647_a_at							I	I	I
IFI16	1419603_at, 1452349_x_at					I		D		
IFI202B	1421551_s_at, 1457666_s_at				I	I	I			
IFIH1	1426276_at					I		D		
IFNAR2	1451462_a_at									I
IGFBP4	1421992_a_at, 1423756_s_at, 1423757_x_at, 1437405_a_at, 1437406_x_at								D	D
IGFBP5	1422313_a_at, 1452114_s_at					D	D		D	D
IGFBP7	1423584_at, 1423585_at					D			I	I
IKBKG	1454690_at									I
IKIP	1429065_at, 1429219_at								I	I
IL15	1418219_at								I	
IL15RA	1448681_at									I
IL18	1417932_at								I	I
IL1RL1	1422317_a_at									I
IL6	1450297_at	I							I	I
INHBA	1422053_at							I	I	I
INPP1	1418045_at, 1442073_at								I	
IRF8	1416714_at, 1448452_at						I			I
IRS1	1423104_at	D						I	I	I
ITGA5	1423267_s_at			D						
ITGB5	1417533_a_at, 1417534_at, 1456195_x_at					D	D			
ITM2B	1417999_at, 1418000_a_at								I	I
ITPR3	1417297_at								I	I
JMJD6	1420056_s_at, 1454109_a_at								D	D
JUN	1417409_at, 1448694_at							D	D	D
KAT2B	1434037_s_at, 1450821_at								I	I
KAT5	1433980_at, 1433981_s_at									D
KIF1B	1455182_at					I				
KITLG	1415855_at, 1448117_at					I				I
KLF10	1416029_at								I	I
KLF4	1417394_at, 1417395_at								I	I
KLF6	1418280_at, 1427742_a_at, 1447448_s_at	D	D	D	I					
LAMP2	1416344_at								I	I
LCN2	1427747_a_at							I		I
LDLR	1421821_at						D		I	I
LGALS3	1426808_at									I
LGALS3BP	1448380_at								I	I
LGALS8	1422662_at								I	
LIF	1421207_at									I
LIMS1	1418232_s_at			D						
LMNA	1421654_a_at, 1425472_a_at, 1457670_s_at			D		D	D	I	I	I
LPAR1	1426110_a_at, 1448606_at					D	D			I
LRIG1	1434210_s_at, 1449893_a_at								I	I
LTBR	1416435_at			D						
MAOA	1428667_at							I	I	I
MAP2K3	1451714_a_at									I
MAP3K12	1438908_at	I								
MAP3K4	1459800_s_at								I	I
MAP3K7	1419988_at									I
MAPK3	1427060_at			D						
MAPK8	1420932_at									D
MAPKAP1	1417284_at								I	I
MAX	1423501_at						D	D		D
MCF2L	1434140_at			D						
MCL1	1416880_at			D						
MCM2	1448777_at, 1423605_a_at, 1427718_a_at							D	D	D
MDM2	1427718_a_at	I		I		I	I		I	I
MED1	1450402_at			I						
MEF2A	1427186_a_at, 1452347_at				I					
MET	1422990_at, 1434447_at								I	I
MFGE8	1420911_a_at									I
MGP	1448416_at					D	D	I		I
MGST1	1415897_a_at							I	I	I
MMP2	1416136_at									I
MMP3	1418945_at							I		I
MPG	1417571_at, 1417572_at								I	I
MT1E	1428942_at					D		I	I	I
MT1F	1422557_s_at						D	I		
MTMR6	1425485_at									I
MTPN	1437457_a_at				I					
MX1	1451905_a_at							D	I	
MYC	1424942_a_at			D						
MYO6	1433942_at									I
NAMPT	1417190_at							D		D
NCAM1	1426864_a_at									I
NCAPG2	1417926_at					I				
NDRG1	1420760_s_at, 1423413_at, 1450976_at, 1456174_x_at							D	D	D
NDST1	1422044_at, 1460436_at			D				D		
NDUFAF4	1427997_at			I						
NDUFV2	1428179_at, 1452692_a_at									I
NEDD9	1422818_at									I
NEK2	1417299_at, 1437580_s_at								I	I
NEK6	1423596_at, 1425850_a_at							I		I
NFAT5	1438999_a_at, 1439805_at			D			I		I	I
NFIL3	1418932_at			D						
NFKB1	1427705_a_at									I
NFKB2	1425902_a_at						I			
NFKBIA	1420088_at, 1438157_s_at, 1448306_at, 1449731_s_at							I	I	I
NFKBIZ	1417483_at, 1448728_a_at, 1457404_at							I	I	I
NGF	1419675_at			D						
NME1	1424110_a_at									D
NOD1	1454733_at								I	I
NOTCH2	1455556_at						D			
NP	1416530_a_at, 1453299_a_at								I	I
NQO1	1423627_at								I	I
NQO2	1449983_a_at, 1455590_at								I	I
NR2F1	1418157_at						D			
NR3C1	1421867_at, 1457635_s_at, 1460303_at								I	I
NR4A1	1416505_at			D						
NRF1	1434627_at									D
NRP1	1418084_at									I
NT5C3	1451050_at								I	I
NTRK3	1433825_at				D	D	D			
NUAK2	1429049_at									I
NUPR1	1419665_a_at						I			
OAS1	1424775_at								I	
OAS1B	1425119_at							D		
OAS3	1425374_at							D	I	I
ODC1	1437711_x_at								D	
OSGIN1	1424022_at			D						
P2RX4	1425525_a_at, 1452527_a_at								I	I
P2RX7	1439787_at								I	
PA2G4	1420142_s_at, 1423060_at, 1435372_a_at			D					D	D
PAFAH1B1	1460199_a_at							D		D
PAK1	1420980_at, 1450070_s_at			D						I
PAK3	1435486_at, 1437318_at			D		I				
PALLD	1427228_at, 1433768_at			D						I
PARK7	1416526_a_at, 1456194_a_at								D	D
PARVA	1431375_s_at								I	I
PARVB	1438672_at									I
PAWR	1426910_at			D						I
PCNA	1417947_at								D	D
PDCD2	1423534_at									D
PDGFRA	1421917_at					D	D			
PDGFRB	1417148_at, 1436970_a_at			D	D	D	D			
PEA15	1416407_at									I
PHLDA1	1418835_at							I	I	I
PIK3CA	1460326_at					I				
PIK3R2	1418463_at									I
PITPNA	1423282_at, 1423283_at								I	I
PKN2	1437295_at, 1437296_at					I				
PLAC8	1451335_at								I	I
PLAT	1415806_at								I	I
PLAUR	1452521_a_at									I
PLD1	1437113_s_at								I	I
PLD2	1417237_at								I	
PLEKHF1	1424671_at								I	I
PLK1	1448191_at			D				I	I	I
PLK3	1434496_at								I	I
PLSCR1	1429527_a_at, 1453181_x_at								I	I
PLSCR3	1449020_at								I	
PMEPA1	1422706_at, 1452295_at			D		D	D			
PML	1448757_at, 1456103_at							D	I	
PNKP	1416378_at								I	I
PNPT1	1452676_a_at							D		
POLK	1449483_at								I	I
PPID	1417057_a_at								D	D
PPM1A	1429501_s_at, 1451943_a_at									D
PPM1F	1454934_at									I
PPP1R13L	1459592_a_at			D						
PPP1R15A	1448325_at								D	D
PPP2R2A	1437730_at, 1453260_a_at							D		D
PRDX5	1416381_a_at									I
PRKAR2B	1438664_at, 1456475_s_at									I
PRKCA	1450945_at									I
PRKD1	1447623_s_at									I
PRMT2	1416844_at								I	
PRPF19	1460633_at						D			
PRR13	1423686_a_at						I		I	I
PSENEN	1415679_at									D
PSIP1	1417166_at, 1460403_at				I	I	I			
PSMG2	1425373_a_at, 1448212_at									D
PTGR1	1417777_at							I	I	I
PTGS1	1436448_a_at									I
PTGS2	1417262_at, 1417263_at							I	I	I
PTMA	1423455_at								D	D
PTPN1	1438670_at			D						
PTPRA	1425340_a_at								I	I
PTPRE	1418540_a_at								I	
PTPRG	1434360_s_at					D	D			
PTRH2	1451845_a_at							D	D	D
PTTG1	1419620_at, 1424105_a_at, 1438390_s_at							I	I	I
PXN	1424027_at, 1456135_s_at								I	I
QARS	1423712_a_at, 1456726_x_at								I	I
QKI	1417073_a_at, 1425597_a_at, 1429318_a_at, 1451179_a_at						D	D	D	D
RABGGTB	1419553_a_at									I
RAD18	1451928_a_at					I				
RAD21	1416162_at			D						
RAD54L	1450862_at					I			I	I
RALB	1417744_a_at									I
RARG	1419415_a_at, 1419416_a_at			D						
RASA1	1426476_at, 1426477_at									I
RASSF1	1441737_s_at, 1448855_at						I			
RASSF5	1422637_at									I
RB1	1417850_at					I				
RBBP4	1434892_x_at, 1454791_a_at, 1454875_a_at							D	D	D
RBBP6	1425114_at									D
RBL1	1424156_at, 1425166_at							D		D
RBP1	1448754_at							I	I	I
RCAN2	1421425_a_at									I
RECK	1450784_at								I	
RFC1	1418342_at, 1449050_at, 1451920_a_at				I	I	I			
RFK	1415737_at, 1416230_at								D	D
RFWD2	1426913_at								I	
RGS3	1425296_a_at, 1425701_a_at							I	I	I
RIPK1	1419508_at, 1449485_at								I	I
RIPK2	1450173_at									I
RNF34	1415791_at			I						
ROCK1	1423444_at, 1423445_at						I			I
RPS3	1435151_a_at			I						
RPS3A	1422475_a_at			I				I		
RPS6KB1	1454956_at					I				
RRAS	1418448_at								I	
RRAS2	1417398_at									I
RRM2B	1437476_at								I	
RTN4	1421116_a_at, 1452649_at					D				I
S100A1	1417421_at, 1419814_s_at								I	I
S100A10	1416762_at, 1456642_x_at							I	I	I
S100A4	1424542_at					D	D	I	I	I
S100A6	1421375_a_at							I		I
S1PR1	1423571_at			D					I	I
S1PR2	1428176_at			D						
S1PR3	1438658_a_at									I
SAT1	1420502_at									I
SCARB1	1416050_a_at, 1437378_x_at, 1455820_x_at								I	I
SDC1	1415943_at, 1415944_at, 1437279_x_at					D	D			I
SDC4	1448793_a_at									I
SEMA3A	1449865_at									I
SENP1	1424330_at									D
SERBP1	1437280_s_at			I						
SERPINE1	1419149_at					D	D	I	I	I
SERPINE2	1416666_at									I
SERPINF1	1416168_at, 1453724_a_at						D			
SFRP1	1448395_at									I
SFRP2	1448201_at					D				
SFRS5	1423130_a_at			I						
SGK1	1416041_at							I	I	I
SGMS2	1428663_at, 1429029_at									I
SGPL1	1415892_at						D			
SH3BP5	1421922_at, 1421923_at								I	I
SH3GLB1	1418011_a_at, 1418012_at			D					I	
SH3KBP1	1431592_a_at, 1460337_at								I	I
SHISA5	1423986_a_at, 1437503_a_at								I	I
SHPRH	1452261_at					I				
SIRT7	1424238_at								I	
SKIL	1452214_at									I
SLC25A24	1427483_at, 1452717_at								I	I
SLC2A1	1426599_a_at, 1434773_a_at				D					I
SLC7A11	1420413_at								I	
SLK	1425977_a_at, 1449336_a_at					I				
SMN1	1426596_a_at								D	D
SMNDC1	1429043_at								D	
SNRPE	1451294_s_at								D	D
SOCS3	1416576_at, 1455899_x_at, 1456212_x_at			D				I	I	I
SOD2	1417193_at, 1448610_a_at									I
SOD3	1417633_at								I	I
SORBS2	1437197_at							I	I	I
SOX4	1419155_a_at, 1419156_at, 1419157_at, 1433575_at, 1449370_at					D	D	D	D	D
SP1	1418180_at, 1454852_at								D	D
SPP1	1449254_at					D	D	I	I	I
SRGN	1417426_at								I	I
STAT1	1420915_at, 1450033_a_at, 1450034_at							D	D	D
STAT5A	1421469_a_at, 1450259_a_at								I	I
STAT6	1426353_at								I	I
STK24	1426248_at						D			
STMN1	1415849_s_at, 1448113_at								D	D
STX8	1418089_at								I	I
SULF1	1436319_at, 1438200_at									I
TACC3	1417450_a_at, 1436872_at, 1455834_x_at								I	I
TADA3L	1417467_a_at									I
TAX1BP1	1420174_s_at, 1448399_at					I				I
TCF12	1427670_a_at									D
TCF4	1416724_x_at				I					
TCF7	1433471_at									I
TENC1	1452264_at			D						I
TERF1	1418380_at									I
TFAP2A	1421996_at, 1426048_s_at						D			D
TGFB1	1420653_at								I	I
TGFB1I1	1418136_at									I
TGFB2	1450922_a_at								I	I
TGFBR2	1425444_a_at, 1426397_at								I	I
TGFBR3	1433795_at					D				I
THBS1	1421811_at, 1450377_at, 1460302_at			D				I	I	I
THBS2	1422571_at, 1447862_x_at, 1450663_at			D		D	D			I
TIAL1	1421148_a_at							D		
TIMP1	1460227_at								I	I
TIMP2	1420924_at, 1433662_s_at, 1450040_at, 1454677_at, 1460287_at							I	I	I
TIMP3	1419088_at, 1419089_at, 1449334_at, 1449335_at					D			I	I
TLR1	1449049_at									I
TLR3	1422781_at, 1422782_s_at					I		D	I	I
TLR4	1418163_at									I
TMEM173	1427911_at, 1447621_s_at						I		I	I
TMSB10	1417219_s_at, 1436902_x_at, 1437185_s_at							I		I
TMSB4X	1415906_at							I	I	I
TNC	1416342_at, 1456344_at					D				I
TNFAIP3	1433699_at								I	I
TNFAIP8	1416950_at								I	I
TNFRSF12A	1418571_at, 1418572_x_at							I	I	I
TNFRSF19	1425212_a_at								I	
TNFRSF1A	1417291_at			D						
TNKS2	1447522_s_at								I	I
TOP1	1423474_at					I				
TOP2A	1454694_a_at					I	I			
TOPBP1	1452241_at					I				I
TOPORS	1417754_at						I			
TP53	1426538_a_at, 1427739_a_at									D
TP53BP2	1433937_at, 1433938_at							D		D
TP53INP1	1416926_at, 1416927_at	I		I		I	I		I	I
TPD52L1	1418412_at									I
TPM1	1423049_a_at, 1423721_at								I	I
TPP1	1434768_at								I	I
TRAF3IP2	1448508_at									I
TRAF7	1424320_a_at								I	I
TRIAP1	1460702_at									D
TRIB2	1426640_s_at					D	D			
TRIB3	1426065_a_at, 1456225_x_at							D	D	D
TRIM27	1438376_s_at, 1456375_x_at									D
TSC2	1452105_a_at									I
TSLP	1450004_at								I	I
TSPO	1416695_at, 1438948_x_at, 1456251_x_at								I	I
TTK	1449171_at					I			I	I
TXN	1416119_at							I		
TXNDC17	1423034_at, 1423035_s_at, 1439184_s_at								I	I
TXNIP	1415996_at, 1415997_at					I		D		D
UBA7	1426971_at								I	I
UBE2C	1452954_at							I	I	I
UBR4	1454668_at							D		
UNG	1425753_a_at					D	D	D	D	D
UTP11L	1429485_a_at									I
UXT	1418986_a_at									D
VCAM1	1415989_at, 1436003_at, 1448162_at, 1451314_a_at								I	I
VCAN	1427256_at									D
VCL	1416156_at, 1416157_at								I	I
VDR	1418175_at, 1418176_at									I
VHL	1434708_at							D		D
WEE1	1416773_at									D
WFS1	1448411_at									D
WISP1	1448593_at, 1448594_at								I	I
WRN	1425982_a_at								I	
WTAP	1454805_at							D		D
WWOX	1416334_at					D	D			
XAF1	1443698_at						I			
XBP1	1420886_a_at, 1437223_s_at					D				
XDH	1451006_at								I	
XPA	1460725_at									I
XRCC2	1455335_at									D
XRCC4	1424601_at								D	D
XRCC6	1417437_at								D	D
YARS	1460638_at									D
YWHAE	1435702_s_at, 1438839_a_at									D
YY1	1435824_at, 1457834_at			D					I	I
ZFP36	1452519_a_at			D					I	
ZFP36L2	1437626_at					D	D			I
ZMAT3	1449353_at			I					I	I
ZNF148	1418381_at, 1449068_at, 1449069_at					I	I			
ZNF622	1438000_x_at								D	D
ZYX	1417240_at							I	I	I

**Table 2:** Differential expression of apoptosis-related genes which have direct upstream or downstream relationship with p53 in each treatment group [irradiation (Gy), cisplatin (CDDP), or combination of both (Gy + CDDP)] when compared to nontreated control cells at 3 h, 24 h, and 72 h after treatment.

3 hours							

Symbol	Gy	CDDP	Gy+CDDP	Symbol	Gy	CDDP	Gy+CDDP
CCNG1	I		I	KLF6	D	D	D
CDKN1A	I		I	CCND1	D		
MDM2	I		I	CTCF	D		
TP53INP1	I		I	IRS1	D		
BTG2	I			ATF3			D
DDX5	I			AURKA			D
GDF15	I			BID			D
IL6	I			CCNB1			D
C11ORF82			I	CEBPB			D
CASP3			I	DDR1			D
CASP6			I	ETS1			D
CRYAB			I	FHL2			D
HSP90AA1			I	HBEGF			D
MED1			I	HIPK1			D
ZMAT3			I	HSPB1			D
				MAPK3			D
				MCL1			D
				MYC			D
				NR4A1			D
				OSGIN1			D
				PLK1			D
				PMEPA1			D
				PPP1R13L			D
				THBS1			D
				THBS2			D
				YY1			D

24 hours							

Symbol	Gy	CDDP	Gy+CDDP	Symbol	Gy	CDDP	Gy+CDDP

CCNG1	I	I	I	APEX1		D	D
CDKN1A	I	I	I	BHLHE40		D	D
EIF2AK2	I	I	I	BRE		D	D
RFC1	I	I	I	PMEPA1		D	D
BUB1		I	I	S100A4		D	D
CCAR1		I	I	SERPINE1		D	D
CKAP2		I	I	SPP1		D	D
HSP90AA1		I	I	THBS2		D	D
MDM2		I	I	WWOX		D	D
TOP2A		I	I	SLC2A1	D		
TP53INP1		I	I	BTG1		D	
ZNF148		I	I	CAV1		D	
KLF6	I			CEBPB		D	
BLM		I		TIMP3		D	
BRCA1		I		ATP1A1			D
HMMR		I		CDK4			D
IFI16		I		CDKN2A			D
RAD54L		I		CDKN2C			D
RB1		I		GSTM5			D
TOP1		I		ID2			D
TOPBP1		I		TFAP2A			D
TTK		I					
BTG2			I				
C11ORF82			I				
FUBP1			I				
NFKB2			I				
NUPR1			I				
TOPORS			I				

72 hours							

Symbol	Gy	CDDP	Gy+CDDP	Symbol	Gy	CDDP	Gy+CDDP

ANXA1	I	I	I	LGALS3			I
AURKA	I	I	I	LIF			I
BUB1B	I	I	I	MAP2K3			I
CAV1	I	I	I	MMP2			I
CCNB1	I	I	I	MYO6			I
CCND1	I	I	I	NFKB1			I
CCNG1	I	I	I	PLAUR			I
CDC20	I	I	I	PRKCA			I
CDKN1A	I	I	I	PTGS1			I
CLU	I	I	I	SAT1			I
DDIT4	I	I	I	SERPINE2			I
EGR1	I	I	I	SLC2A1			I
FOSL1	I	I	I	SOD2			I
GLIPR1	I	I	I	TADA3L			I
HSPB1	I	I	I	THBS2			I
IER3	I	I	I	TOPBP1			I
INHBA	I	I	I	TSC2			I
IRS1	I	I	I	VDR			I
NFKBIA	I	I	I	FEN1	D	D	D
PHLDA1	I	I	I	GADD45A	D	D	D
PLK1	I	I	I	JUN	D	D	D
PTGS2	I	I	I	MCM2	D	D	D
PTTG1	I	I	I	NDRG1	D	D	D
S100A4	I	I	I	STAT1	D	D	D
SERPINE1	I	I	I	ATF3	D		D
SGK1	I	I	I	PPP2R2A	D		D
SPP1	I	I	I	TP53BP2	D		D
THBS1	I	I	I	CDK4		D	D
TMSB4X	I	I	I	FUBP1		D	D
UBE2C	I	I	I	GSK3B		D	D
ZYX	I	I	I	HMGB1L1		D	D
BCL3	I		I	HSPA5		D	D
MMP3	I		I	PARK7		D	D
S100A6	I		I	PCNA		D	D
ABCB1B		I	I	PPP1R15A		D	D
AKAP12		I	I	SMN1		D	D
ATM		I	I	SP1		D	D
BID		I	I	STMN1		D	D
BIRC5		I	I	XRCC6		D	D
BUB1		I	I	DAXX	D	I	
CCNA2		I	I	MX1	D	I	
CCND3		I	I	PML	D	I	
CDC25C		I	I	E2F1	D		
CKAP2		I	I	IFI16	D		
CRYAB		I	I	BRE			D
CTSD		I	I	CDC6			D
DDR1		I	I	CHEK1			D
DHCR24		I	I	COPS5			D
EZR		I	I	CTCF			D
FHL2		I	I	DDIT3			D
FOS		I	I	DUT			D
FOXM1		I	I	ELAVL1			D
HMMR		I	I	HOXA7			D
IL6		I	I	HUWE1			D
KAT2B		I	I	KAT5			D
KLF4		I	I	MAPK8			D
MDM2		I	I	NME1			D
MET		I	I	RBBP6			D
NEK2		I	I	TFAP2A			D
NQO1		I	I	TP53			D
NQO2		I	I	VCAN			D
NR3C1		I	I				
PLK3		I	I				
PTPRA		I	I				
RAD54L		I	I				
S100A1		I	I				
SHISA5		I	I				
TACC3		I	I				
TGFB2		**I**	**I**				
TIMP3		**I**	**I**				
TP53INP1		**I**	**I**				
TTK		**I**	**I**				
YY1		**I**	**I**				
ZMAT3		**I**	**I**				
TXN	**I**						
GDF15		**I**					
GSTM1		**I**					
RFWD2		**I**					
RRM2B		**I**					
WRN		**I**					
AFP			**I**				
AHR			**I**				
AP2A2			**I**				
BHLHE40			**I**				
C11ORF82			**I**				
CASP6			**I**				
CAT			**I**				
CDK8			**I**				
CENPF			**I**				
CFLAR			**I**				
CSF2			**I**				
CX3CL1			**I**				
EPHA2			**I**				
ERCC3			**I**				
ERCC5			**I**				
ETS1			**I**				
FAS			**I**				
FASN			**I**				
GPI			**I**				
HK2			**I**				
HSP90AB1			**I**				
ID1			**I**				
